# Bleeding Complication of Triple Therapy of Rivaroxaban, Prasugrel, and Aspirin: A Case Report and General Discussion

**DOI:** 10.1155/2014/293476

**Published:** 2014-03-11

**Authors:** Dane D. Gruenebaum, Ahmad Alsarah, Osama Alsara, Heather Laird-Fick

**Affiliations:** Department of Internal Medicine, Michigan State University, B-301 Clinical Center, East Lansing, MI 48824, USA

## Abstract

Hemorrhagic side effects are the bane of oral anticoagulation. Despite careful selection of medications and close monitoring, some adverse events are unavoidable. The available literature about the risks of triple oral anticoagulation therapy versus dual antiplatelet therapy does not address all of the medication combinations currently available. This report describes a patient with atrial fibrillation and recent stent placement who developed severe, recurrent epistaxis on aspirin, prosugrel, and rivaroxaban. We believe this is the first case report of severe bleeding with this combination, and it may help provide insights into the risk for other patients.

## 1. Introduction

Several options, with well-described bleeding risks, exist for the chemoprophylaxis of thrombotic complications of either atrial fibrillation or coronary stents in isolation. However, therapy is less clear when the two conditions are comorbid. The “gold standard” therapies differ for each and provide suboptimal risk reduction for the other condition. For example, the combination of aspirin and a thienopyridine inhibitor is recommended to prevent in-stent thrombosis but is inferior to warfarin or the newer oral anticoagulants for the prevention of stroke in atrial fibrillation. As a result, some clinicians have utilized both gold standard therapies simultaneously, resulting in triple oral antithrombotic therapy. While this strategy theoretically minimizes the risk of thrombotic complications, it may increase the risk of bleeding beyond the benefits gained. The net risk-benefit ratio for patients for patients on triple antithrombotic therapy should drive treatment decisions, yet it is often unclear.

## 2. Case

A 55-year-old Caucasian male presented to the emergency department for one-day history of intractable nausea and vomiting and was found to be in atrial fibrillation with rapid ventricular response. He had a known history of paroxysmal atrial fibrillation but had previously refused warfarin therapy. His past medical history was otherwise significant for congestive heart failure preserved ejection fraction, coronary artery disease treated with a bare metal stent six months prior, diabetes mellitus type 2, hypertension, gout, and chronic kidney disease stage 3 (creatinine clearance 30). His home medications included prasugrel, aspirin atorvastatin, clonidine, furosemide, potassium chloride, amlodipine, insulin, allopurinol, and hydralazine.

His nausea and vomiting were treated with supportive care and he was started on heparin and diltiazem drip for his atrial fibrillation. Cardiology recommended rivaroxaban for thromboembolism prophylaxis as the patient's main objection to warfarin was the need for frequent monitoring and dose adjustment.

Within 72 hours of initiating renally dosed rivaroxaban, the patient experienced recurrent nasal bleeding. During the day the patient had five episodes of mild bleeding that stopped with direct pressure and simple packing. However, that night the patient experienced severe posterior nasal hemorrhage requiring posterior nasal packing. Aspirin was discontinued. When the packing was removed five days later, bleeding recurred. Cardiology reevaluated the patient's antithrombotic regimen, discontinuing rivaroxaban and switching prasugrel to clopidogrel. After 24 hours without oral rivaroxaban or recurrence of hemorrhage, the patient was deemed stable for discharge.

## 3. Discussion

For decades, antithrombotic therapies have been effectively used to decrease complications associated with medical conditions such as atrial fibrillation, mechanical heart valves, deep venous thrombosis, and intravascular stents. An agent's mechanism of action (inhibition of the coagulation or platelet aggregation cascades) makes it more effective for specific cardiac diseases rather than others. For instance, aspirin and clopidogrel dual therapy is considered the standard of care to prevent stent thrombosis [1] but is inferior to oral anticoagulants for preventing stroke in high-risk patients with atrial fibrillation [2]. Similarly, oral anticoagulants reduce stroke risk in patients with atrial fibrillation [3, 4] but are not effective as monotherapy after stent [5].

About 5–7% of patients undergoing percutaneous coronary interventions (PCI) have atrial fibrillation (AF) or other indications for chronic oral anticoagulant therapy [6]. The combination of antithrombotic regimens into a “triple therapy” seems like an attractive option for preventing both stroke and in-stent thrombosis but must be balanced against a bleeding risk which is less well understood.

The most studied oral triple antithrombotic therapies are aspirin, warfarin, and clopidogrel. A meta-analysis of nine trials included 1,996 participants taking either dual antiplatelet therapy (aspirin and clopidogrel) or triple therapy (warfarin, aspirin, and clopidogrel) has reported that triple therapy was more efficacious in reducing the occurrence of cardiovascular events and mortality compared with dual antiplatelet therapy. However, there was significantly more major bleeding with triple antithrombotic therapy in the first 6 months (OR, 2.12; 95% CI, 1.05–4.29; *P* = 0.04) [7].

Generally, bleeding events with triple therapy have been found to be 3.7 times higher than that with warfarin alone and 4 times than with aspirin monotherapy [8, 9]. Similarly, adding warfarin to dual antiplatelet therapy increases the relative risk for bleeding to 2.2 at 30 days [10], and the yearly incidence of bleeding will be 12% compared to 3.7% for only aspirin plus clopidogrel [9]. Interestingly, combining warfarin and only clopidogrel reduces bleeding complications without increasing thrombotic events when compared to triple therapy [11].

Lack of large randomized control trials in this area has resulted in divergence in medical society recommendations. American College of Cardiology/American Heart Association guidelines recommended that high-risk patients with concurrent AF and coronary stents receive therapy of warfarin and clopidogrel for 1–12 months depending on the type of stent and then warfarin as monotherapy [12]. On the other hand, the 2012 American College of Chest Physicians (ACCP) guideline on antithrombotic therapy for atrial fibrillation stratified patients depending on their risk of stroke, using CHADS2. For patients with CHADS2 score of greater than 1 and recent stent placement, ACCP recommends triple therapy for one (bare metal stent) or 3–6 (drug-eluting stent) months, followed by warfarin and a single antiplatelet drug for 12 months, and then warfarin monotherapy. For patients with CHADS2 score of 0 or 1, the recommendation is dual antiplatelet therapy for 12 months and then warfarin [13]. In clinical practice, physicians' strategies broadly vary between triple therapy, dual antiplatelet therapy, and oral anticoagulation with aspirin. The decision is usually influenced by the anticipated bleeding risk of combined pharmacotherapy [14].

Most of the research on triple therapy, as well as current guidelines, has included warfarin. There appears to be a paucity of information involving the utilization of newer agents in triple therapy. Rivaroxaban is a direct factor X inhibitor that has demonstrated efficacy in preventing stroke and systemic embolism in patients with atrial fibrillation but did not remarkably reduce the rates of bleeding when compared to warfarin [15]. It cannot be monitored or reversed, unlike warfarin. There are two studies in which triple therapy including rivaroxaban was compared to dual antiplatelet therapy in patients with STEMI, NSTEMI, or unstable angina. The ATLAS ACS-TIMI 46 is a Phase-2 study that included 3491 patients who were randomized to three groups: placebo, rivaroxaban 5–20 mg daily, or rivaroxaban 5–10 mg twice daily. In this study, rivaroxaban 20 mg was not associated with a decreased rate in the combination of death, MI, stroke, or severe recurrent ischemia requiring revascularization but resulted in an increased risk of bleeding (HR 5.06 [3.45–7.42]; *P* < 0.00015) compared to placebo. Bleeding risk was increased in a dose-dependent manner in relation to total daily doses of rivaroxaban [16]. The next study, ATLAS ACS2-TIMI 51, was a Phase-3 study in which rivaroxaban 2.5 mg daily, rivaroxaban 5 mg daily, or placebo was added to dual antiplatelet therapy in 15,526 patients with STEMI, NSTEMI, or unstable angina. This study found that triple therapy increased the rates of major bleeding (2.1% versus 0.6%; *P* < 0.001) and intracranial hemorrhage (0.6% versus 0.2%; *P* = 0.009), without a significant increase in fatal bleeding (0.3% versus 0.2%; *P* = 0.66) compared to placebo but with a significant improvement in cardiovascular mortality, MI, or stroke [17]. Although both two doses of rivaroxaban reduced cardiovascular events in patients with recent acute coronary syndromes treated with antiplatelet therapies, the 2.5 mg dose was associated with lower mortality and fewer bleeding complications than the 5 mg dose, and it seemed to provide a more balance of efficacy and safety in those patients [18].

Many factors increased our patient's risk of bleeding. Prasugrel results in greater platelet inhibition and more bleeding compared to clopidogrel [19, 20]. Advanced chronic kidney disease may increase the bleeding risk. In the ROCKET AF trial, in which rivaroxaban was compared to warfarin in nonvalvular atrial fibrillation, patients with creatinine clearance <30 mL/min were excluded, and those with a creatinine clearance of 30–49 mL/min received a reduced dose of rivaroxaban (15 mg daily) [15]. To date, there is no published data on the effect of kidney failure on bleeding risk of triple therapy with rivaroxaban.

When the anticipated risk of stroke overweigh the risk of bleeding, and the use of triple therapy becomes mandatory, many strategies can be considered to decrease bleeding risk, such as using the lower effective dose of aspirin [10, 21] and adding proton pump inhibitor as a prophylaxis of gastrointestinal bleeding [10, 22]. Utilizing bare metal stents to minimize time on triple therapy may be another strategy to prevent bleeding events, but at this point it is based on insufficient evidence.

## 4. Conclusion

We believe this is the first case report of bleeding complications involving this combination of medications. Care should be taken in selecting a regimen most appropriate for the patient, remembering that there are no methods for monitoring newer oral anticoagulants. This is especially true in patients with other comorbidities that increase bleeding risk, such as chronic kidney disease.
